# Open surgical treatment of a 34-year-old arteriovenous fistula of the lateral plantar artery after failed coil embolization

**DOI:** 10.1016/j.jvscit.2026.102335

**Published:** 2026-05-26

**Authors:** Elena Streck, Valerian Zhghenti, Christoph Düber, Savvas Savvidis, Achim Neufang

**Affiliations:** a Department of Cardiac and Vascular Surgery, University Medical Centre Mainz, Johannes Gutenberg University, Mainz, Germany; b Department of Radiology, University Medical Centre Mainz, Johannes Gutenberg University, Mainz, Germany; c Department of Internal Medicine I, Klinikum Hanau, Hanau, Germany

**Keywords:** Traumatic arteriovenous fistula, AVF, Endovascular embolization, Lateral plantar artery, Surgical fistula closure

## Abstract

Trauma-induced arteriovenous fistulas of the plantar artery are exceedingly rare. Although they may initially remain asymptomatic, progressive shunt flow can, over time, result in venous hyperperfusion and congestion. Definitive treatment requires closure of the fistula to restore physiological venous circulation. Endovascular embolization is the primary, minimally invasive treatment and is effective in achieving thrombosis and symptom control in many cases. In the present case, endovascular coil embolization was insufficient, and surgical closure of a chronic arteriovenous fistula of the lateral plantar artery proved to be a reliable and promising treatment alternative.

A trauma-induced arteriovenous fistula (AVF) is an abnormal direct connection between an artery and an adjacent vein that develops after injury.^1^ Exposure of the vein to high arterial pressure results in chronic hemodynamic overload and progressive weakening of the venous wall, which in turn promotes the formation of a pseudoaneurysm as a vascular abnormality characterized by disruption of one or more layers of the vessel wall.^1^ More than 50% of traumatic AVFs occur in the lower extremities, and approximately one-third occur in the femoral vessels; 15% are in the popliteal vessels.^2^ Such a formation in a pedal artery is very unusual.^3^

These fistulas can lead to significant hemodynamic changes, including turbulent blood flow, venous hypertension, and, in severe cases, high-output cardiac failure.^2^ Untreated traumatic AVFs may cause edema of the limbs, ischemia, or long-term vascular complications.^1^ Time to diagnosis of the AVFs or trauma-induced pseudoaneurysm may be variable and range from 3 days to several years after trauma.^3^ Imaging modalities such as Doppler ultrasound examination, computed tomography angiography, or conventional angiography are most commonly recommended for the diagnosis.^3^

Although endovascular techniques such as coil embolization are often used for newly diagnosed or small post-traumatic AVFs, chronic or complex fistulas with associated venous aneurysms are most commonly treated with open surgical excision, which provides definitive closure and symptom resolution.^4^^,^^5^

We herein report a case of a patient with the surgical treatment of a chronic AVF of the lateral plantar artery after failed coil embolization. Written informed consent was obtained from the patient for publication of this case and accompanying images.

## Case report

A 51-year-old man presented to our vascular surgery outpatient clinic with a post-traumatic plantar AVF and a large venous aneurysm that had undergone multiple treatments with coil embolization. According to his medical history, the patient sustained a penetrating knife injury to the plantar aspect of his left foot extending to the bone at the age of 16. The wound was initially managed with simple suturing. Two years later, he developed persistent pressure and nonspecific pain in the sole of the injured foot. Over the subsequent 25 years, progressive unilateral leg swelling occurred, accompanied by ongoing discomfort and increasing paresthesia of the foot during weight-bearing. Finally, a plantar AVF and a large venous aneurysm were diagnosed when the patient was 47 years old. The venous aneurysm was treated in a minimally invasive manner with coil embolization. Due to persistent symptoms, the procedure was repeated twice. However, foot function continued to deteriorate, as constant pressure in the sole of the foot led to marked impairment of daily activities. On examination, prominently visible veins were observed on the dorsum of the foot, with a palpable thrill upon warming of the overlying skin (Fig 1). Duplex ultrasound examination detected a venous aneurysm with a maximal diameter of 22.5 mm and a flow of 800 mL/min. Angiography demonstrated persistent perfusion of the coiled venous aneurysm via the hypertrophic lateral plantar artery, while perfusion through the anterior tibial artery remained normal (Fig 2).

**Fig 1:**
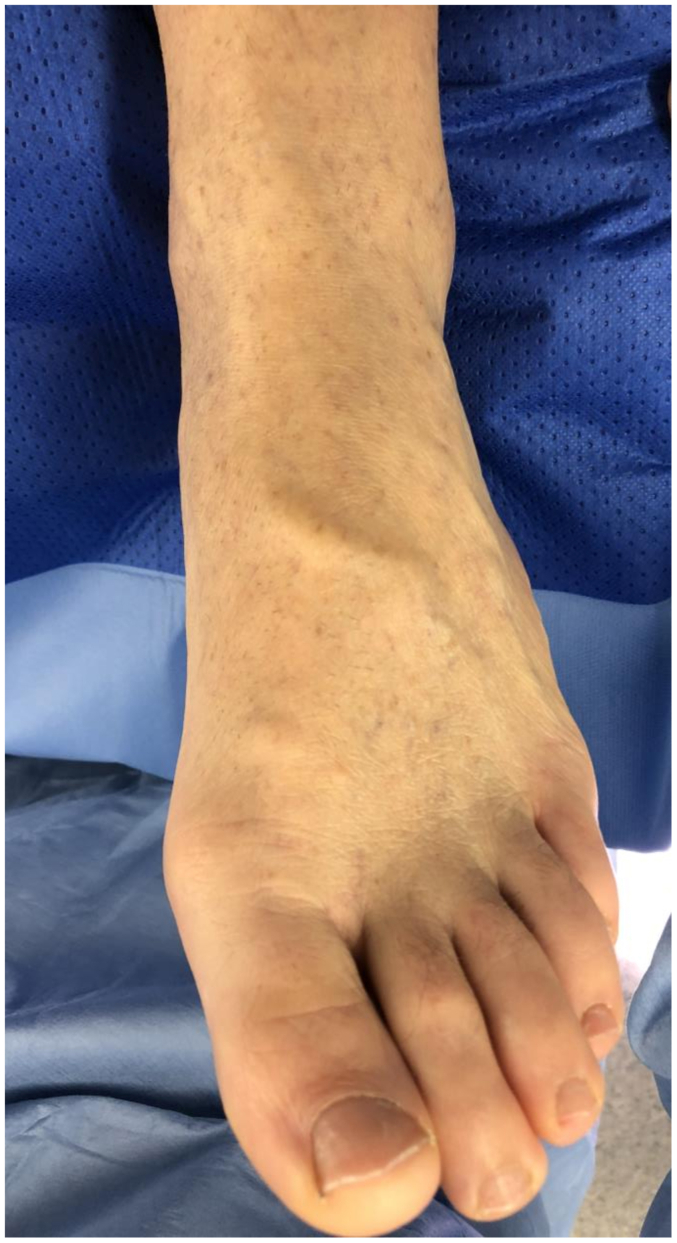
Preoperative clinical examination showing prominently visible dorsal foot veins with a palpable thrill.

**Fig 2:**
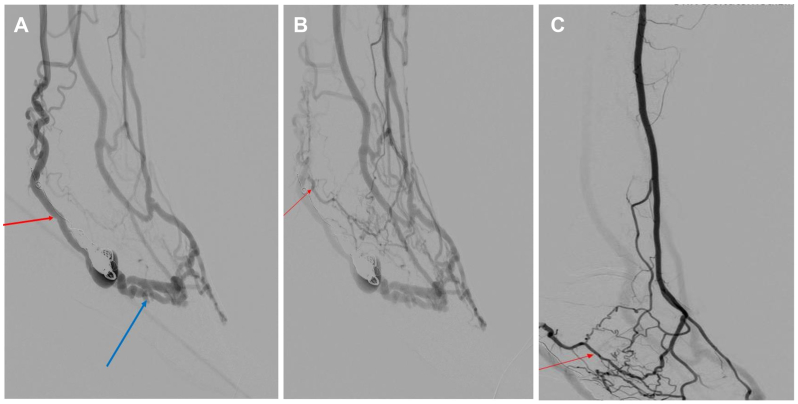
Early phase of selective angiography of hypertrophic posterior tibial artery (*red arrow*) with rapid venous outflow (*blue arrow*) and venous aneurysm with embedded coils **(A)**. Late phase of selective angiography of posterior tibial artery with arterial outflow into the plantar arch (*red arrow*). There are no distal arteries visible **(B)**. Selective angiography of anterior tibial artery with arterial perfusion of the forefoot and plantar arch (*red arrow*) **(C)**.

Given persistent symptoms and incomplete thrombosis, surgical excision of the AVF was indicated. The patient underwent surgical excision of the AVF via an extensive medial approach to the lateral plantar artery under bloodless conditions using an Esmarch bandage. The bandage was removed after wrapping the foot up to the ankle, leaving the proximal portion in place as a tourniquet. The large venous aneurysm was completely resected, including removal of all previously placed coil embolization material, and the arterial connection was closed from within using a simple suture technique (Fig 3).

**Fig 3:**
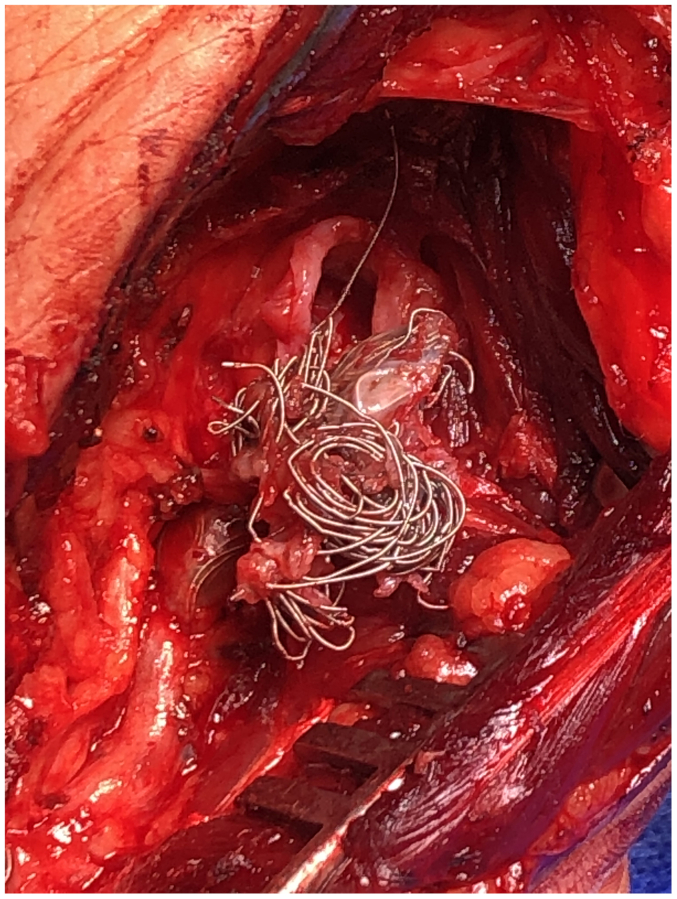
Intraoperative image demonstrating venous aneurysm with placed coil embolization material.

Postoperatively, after primary wound healing and a period of temporary offloading, the patient experienced complete resolution of symptoms with full regression of leg swelling (Fig 4). Computed tomography angiography demonstrated partial preservation of the lateral plantar artery, with no residual aneurysm and maintained foot perfusion via the anterior tibial artery (Fig 5). At 60 months postoperatively, the patient remains symptom free and is able to ambulate without restrictions, including long distances.

**Fig 4:**
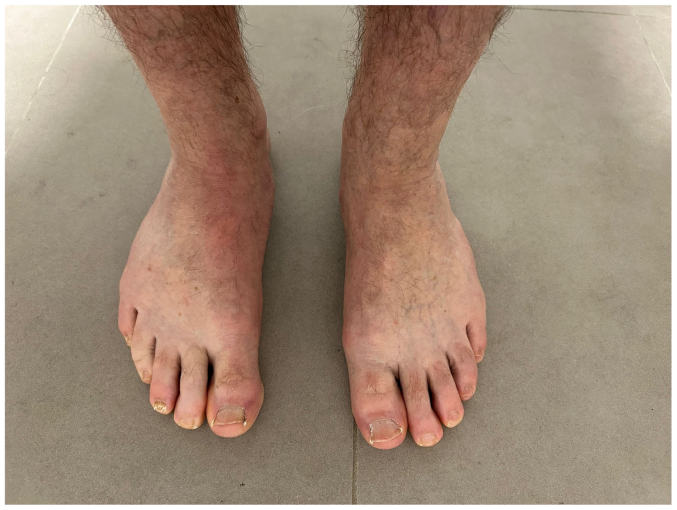
Full regression of leg swelling after the surgery for a 34-year-old arteriovenous fistula of the lateral plantar artery after failed coil embolization.

**Fig 5:**
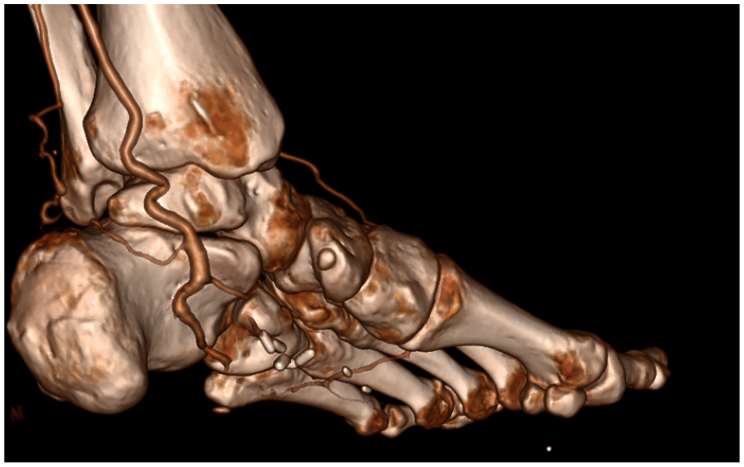
Postoperative computed tomography angiography demonstrating partial preservation of the lateral plantar artery and complete resolution of the venous aneurysm with intact anterior tibial artery perfusion.

## Discussion

Chronic post-traumatic plantar AVFs are exceptionally rare vascular lesions.^3^ A review of the literature demonstrates that chronic post-traumatic plantar AVFs are infrequently reported**,** and most available data come from isolated case reports or small series.^6^, ^7^, ^8^ They can remain asymptomatic for decades and are often only diagnosed when complications such as venous aneurysm formation, progressive limb swelling, pain, or neuropathic symptoms develop.^1^ Our case illustrates the long-term consequences of an untreated or inadequately treated plantar AVF, which, in this patient, became clinically significant over a 34-year period.

Initial endovascular management with coil embolization is generally considered a minimally invasive option for AVFs. Beyond simple coil embolization, additional endovascular strategies have been described, including coil-assisted caging techniques, the use of multilayer stents, and combined distal and proximal embolization approaches. Several technical strategies may improve embolization success in high-flow AVFs. Accurate preprocedural imaging and a detailed understanding of the vascular anatomy are essential. Selective or superselective catheterization close to the fistulous point reduces nontarget embolization and improves occlusion rates.^9^ In high-flow lesions, adjunctive flow-control techniques such as temporary balloon occlusion may improve embolic stability and enable controlled delivery.^10^

Embolic material should be selected according to the angioarchitecture. Coil embolization is effective when the feeding vessel can be safely sacrificed, but requires appropriate oversizing and secure anchoring to prevent migration.^11^ In large or high-flow fistulas, detachable coils or vascular plugs may enhance precision and safety. Combined techniques, using coils as a scaffold followed by liquid embolics such as n-butyl cyanoacrylate or Onyx (Micro Therapeutics) may improve durability.^11^ In selected cases, venous access with complete packing of the draining vein or combined proximal-distal approaches may be advantageous.

Partial treatment, including isolated proximal arterial ligation, should be avoided because it promotes collateralization and recurrence.^12^ Definitive therapy should aim for complete angiographic exclusion of the AVF during the index procedure. However, embolization can be limited in chronic, complex fistulas with large venous aneurysms or when extensive collateral circulation exists.

In this case, repeated embolizations were insufficient to achieve complete thrombosis, resulting in persistent symptoms and progressive functional impairment due to a mass near the weight-bearing region of the foot formed by the coil material. This case highlights the limitations of endovascular therapy in long-standing post-traumatic plantar AVFs and the need for careful patient selection.

Surgical excision remains the definitive treatment in cases where endovascular therapy fails or is unsuitable. The plantar region presents unique anatomical challenges, including confined space, proximity to critical neurovascular structures, and the need to preserve distal perfusion. The integrity of the plantar arch and its communication with the dorsalis pedis artery are essential for maintaining adequate toe perfusion. Because the lateral plantar artery is a key contributor to the deep plantar arch, its occlusion may significantly compromise distal blood flow, depending on the adequacy of collateral supply from the dorsalis pedis system.^13^ This consideration should guide the decision between arterial ligation and preservation. Preoperative selective angiography in our patient demonstrated poor preservation of the distal part of the lateral plantar artery due to massive venous outflow.

In our patient, the use of a simple Esmarch tourniquet allowed safe and bloodless exposure**,** enabling complete resection of the venous aneurysm, explantation of the previously placed coil material, and definitive closure of the AVF. Postoperative imaging confirmed preserved perfusion via the lateral plantar and anterior tibial arteries.

Limitations of this report include its single-case design. The patient remains symptom free and fully ambulatory 60 months after the operation. Long-term monitoring is necessary to detect potential recurrence or late complications. Nonetheless, the successful outcome in this case emphasizes that carefully planned surgical intervention can be curative, even in complex, chronic cases.

## Conclusions

This case contributes valuable insights into both the natural history of untreated plantar AVFs and the technical considerations required for successful surgical management. The application of a simple Esmarch bandage provided safe and uncomplicated access to a complex, chronic plantar fistula, permitting explantation of the previously placed coil material and definitive closure of the AVF. Definitive surgical exclusion, although technically challenging, can achieve complete symptom resolution, preserve limb function, and maintain distal perfusion. Awareness of this rare condition and timely referral to experienced vascular surgeons are essential for optimal outcomes.

## Funding

None.

## Disclosures

None.
